# Attitudes About NIPT Routinisation: A Report from a Qualitative Study of 20 UK Healthcare Professionals

**DOI:** 10.1007/s10728-025-00513-6

**Published:** 2025-02-21

**Authors:** Peter D. Young

**Affiliations:** Ethox Centre, Nuffield Department of Population Health, University of Oxford, Oxford, England

## Abstract

All healthcare professionals (HCPs) have responsibilities to provide information to patients according to the duties found within UK decision-making guidance and with regards to theory about the doctor-patient relationship. While routinisation can be understood in a number of different ways, this paper is concerned with how routines might negatively affect patients in the decision-making process. Therefore, in this manuscript, medical decision making is understood as problematically routine when a medical test or procedure is framed as a standard one and—given the way options are presented—it is implied that someone can decide to use that test or treatment without being given sufficient opportunities to think through their choices. Routinisation, when understood in this way, can affect the quality and amount of information provided to patients and the ways in which patients reflect upon their choices when making decisions. With the introduction of non-invasive prenatal testing (NIPT) into the UK healthcare system, bioethicists have questioned whether this new technology will be routinised and what the implications of routinisation on the decision-making process might be. While there have been numerous studies investigating the views and attitudes of pregnant women who use NIPT, there are fewer studies that look at the views and attitudes of healthcare professionals (HCPs). This study interviewed 20 UK-based HCPs who either offer NIPT or counselled pregnant women on the use of NIPT. One important finding was that many HCPs held the attitude that the NIPT decision-making process had become routine, however there was disagreement about whether routine NIPT was problematic or not. This study provides insights about the context that surrounds decision making for NIPT, and it raises important questions about how NIPT routinisation might be evaluated.

## Introduction

All healthcare professionals (HCPs) have responsibilities to provide information to patients [1–3], and this forms part of the duties found within guidance related to decision making in the UK [4]. The ways in which patients reflect upon their choices when making decisions can be impacted by routinisation. While routinisation can be understood in a number of different ways [5], this paper is concerned with how routines might negatively affect our duties to patients in the decision-making process and with regards to the doctor-patient relationship. There is nothing inherently problematic about routines or the routinisation of new medical technologies, and some peer-reviewed literature indicates that routines might be both beneficial or problematic depending on the situation [6]. However, in her book *The Tentative Pregnancy*, Barbara K. Rothman suggests that medical decision making might be routinised when a medical test or procedure is framed as a standard one and—given the way options are presented—it is implied that someone can decide to use that treatment or test without being given sufficient opportunities to think through the choices [7, p. 51].

According to Rothman’s account, routinisation can impact the amount and quality of information provided to patients, the length of time for the decision-making process, and the quality of reflection about one’s choices. Further, when something is presented in a routine manner, it could be more difficult for a patient to see it as something that they could potentially refuse. This means routinisation may also be connected with consent when it is understood as an informed authorisation [8], and it may also be connected to the way in which autonomy is respected when understood as an agent’s ability to self-legislate moral action [9]. Because of these issues with information and understanding, routinisation might have implications for whether the decision-making process has occurred ethically according to UK guidance on decision making [4]. Thus, investigating whether new technologies are used routinely and whether there is something problematic about certain routines is important when considering the ethical use of that technology.

It will first be important to clarify some of the language that appears in this study about decision making. Some in the philosophy literature have made a distinction between decision making and the decision, especially with regards to shared decision making [10], the framework under which all UK HCPs work [4]. This distinction between decision making and the decision will be adopted here. This study is concerned with the decision-making process that subsumes the offer, and at times, ‘offer’ and ‘decision-making process’ might be used interchangeably. This is because the conversations that occur between the patient and HCP after the offer typically clarifies aspects of the offer.

The impact of routinisation has been described in past studies surrounding prenatal care. Sherwin, for example, questions whether women’s autonomy is respected in the context of some prenatal medical procedures that have become routine, including the introduction of prenatal ultrasound into clinics [11]. She argued that the introduction of ultrasound and its routine use “created a new set of expectations about the experience and customs of being pregnant” [11 p. 102].

In most nations, no laws forbid or mandate ultrasound for pregnant women. No woman is explicitly coerced by her physician or anyone else into accepting ultrasound… Still, there are questions to be asked about the degree of autonomy available to a woman regarding use of this technology [11 p. 104]

Where there are no strong inflection points in the decision-making process, and when a test is offered as a standard procedure, healthcare users might make decisions in accordance with social norms. Like ultrasound, it has been argued that other technologies have become routinised during pregnancy, such as amniocentesis and maternal serum screening [7, 12].

Bioethicists have questioned whether non-invasive prenatal testing (NIPT) could similarly become routinised like those prenatal tests mentioned above. NIPT is a relatively new prenatal test that—by sampling a pregnant woman’s blood—can screen a foetus for Down’s, Edwards’, and Patau’s syndromes as well as other genetic anomalies [13]. NIPT requires a blood draw from the pregnant woman, which can be analysed in a laboratory and used to isolate naturally occurring placental DNA [13]. This process can check for the number of chromosomes and other genetic mutations in the developing foetus.

NIPT was offered in private UK clinics in 2012 for the first time, and the test was integrated into NHS England through their Foetal Anomaly Screening Programme from 2021 [14], although select NHS trusts offered NIPT prior to that 2021 rollout date. Within the NHS, NIPT is offered as a second-tier test to women whose first trimester combined test indicates that the foetus is at high risk for Down’s, Edwards’, or Patau’s syndromes [14]. It is currently offered as a mid-point test between first-tier screening tests and invasive, diagnostic tests. While NIPT is only approved as a screening test for Down’s, Edwards’, and Patau’s syndromes in the NHS, it is possible to access NIPT screening services for a broader range of genetic anomalies in private UK clinics.

Some of the characteristics of NIPT—namely, its safety, accuracy, and non-invasiveness—have important advantages over older prenatal tests [15], and these differences could affect the ethics surrounding its use in a clinical context. NIPT has already been described as “just a blood test” [16, p. 618] and as a “simple blood test” [17, p. 6] in the peer-reviewed literature. According to one study, prenatal tests described as a ‘simple blood test’ had pre-test consultation sessions that lasted no longer than two minutes [12]. Even though a routine decision-making process might be the norm for other screening tests (for example, the combined test), the views of women who used NIPT suggest that a similarly routine process for NIPT could be problematic [18, 19]. It might be useful then to first review the views of both women and healthcare professionals (HCPs) on the specific topic of NIPT routinisation in the section that follows.

There are a number of studies that collected women’s views on the routinisation of NIPT. For example, researchers who conducted qualitative research on UK women’s views about NIPT noted that the perceived ease of the test procedure could lead to the routinisation of the test and the erosion of the informed consent process [18, 19]. Pregnant women noted similar concerns about NIPT being routine, highlighting that it could place pressure on women to take the test, especially when it was offered by a trusted HCP [19]. Another survey of women indicated that it was the non-invasive characteristics of NIPT that might affect informed consent [20]. According to their survey, at least a quarter of the women sampled reported they believed the non-invasiveness of NIPT, when placed in a routine context, might lead to pressure being placed on women to take the test.

Interestingly, a comparative study in the UK has already shown that when NIPT was offered in a routine setting, the quality of informed choice decreased [21]. While it is not entirely clear whether the authors equated a ‘routine clinical setting’ with a ‘routine decision-making process’, it can still be noted that their study reported informed choice decreased from 89% to 75% when NIPT moved from a research environment to a routine prenatal care setting. Where prenatal testing is accepted without being questioned, women could make decisions that are not the result of careful reflection but rather a result of social pressure [22]. This type of uninformed decision making has led women in the past to make decisions that do not align with the preferences they hold [23]. A routine NIPT decision-making process could mean fewer chances to ask for additional information about NIPT and fewer opportunities to refuse the test. This could undermine informed consent by disrespecting women’s autonomy.

While these are valid concerns, recent empirical studies within a Dutch context have indicated that women who received a routine offer of NIPT did not feel pressured to take the test and felt that their choice to take it was in-line with their healthcare values [24–26]. The studies in question were from the views of women in a Dutch context, and they suggest that NIPT routinisation might not be occurring in the Netherlands [24, 26]. If this is true, it might still be the case that the Dutch context does not adequately speak to the UK healthcare context where NIPT is offered via a different clinical pathway structure, healthcare system, and social context. Also, since NIPT is offered as a first-tier test in the Netherlands [24, 26]—which is different from the UK offer where NIPT is offered as a second-tier test [14]—it is not clear how well these results might translate into the UK context. Finally, it is entirely possible that UK HCPs might have a different view about routinisation when compared to the views of women, and those perspectives are worth considering since HCPs make up one half of the HCP–professional relationship.

Few studies have investigated the views of HCPs about the routinisation of NIPT. One study of both women and HCPs in a Japanese context found that both groups were concerned that NIPT might be used without full consideration being given to the test [27]. Interestingly, that specific concern was more pronounced among professionals than women, and some HCPs in the study believed that the counselling provided might not be sufficient for women because NIPT was non-invasive. Another study that interviewed HCPs held the opposite view [28]. HCPs in that study mentioned their belief that the offer and uptake of NIPT can occur during the same consultation, as opposed to being offered on separate days [28]. If clinicians believe this, it is possible they also believe the decision to use NIPT is straightforward, not requiring much deliberation, or perhaps they believe there is not much information worth disclosing. Afterall, NIPT has been described as a simple blood test [16, 17]. Not only is there disagreement within the literature with regards to HCPs’ attitudes and beliefs about NIPT routinisation, there is a dearth of literature in the UK on the topic of NIPT routinisation. A recent paper reported the values of HCPs who offer NIPT in England, and it also discusses non-directiveness and the pressure to test when using NIPT [29]. While the authors of that study mention routinisation as an important consideration, NIPT routinisation was not discussed in depth.

More empirical data is needed to understand whether a routine NIPT decision-making process is occurring in the UK and what the benefits and harms of such an offer might be. This need for more empirical evidence coincides with recent papers published about NIPT routinisation where it was found that more empirical evidence was needed to understand which normative arguments had merit [5, 6].

More research in the UK could shed light on whether HCPs believe that a routine decision-making process for NIPT respects women’s autonomy and what the benefits and harms are of such a process. Whether decision making has occurred ethically rests on the relationship between the patient and HCP. This is a special relationship where both stakeholders need one another to some extent. The patient needs to provide expertise about their preferences, and the HCP provides expertise about medicine. As one half of the relationship, understanding HCPs’ attitudes are important because decision making is a joint activity, being described as a shared activity in the UK healthcare context [4]. Understanding HCPs’ views helps us better understand the healthcare context, and by doing such an analysis, we can see if there are different avenues to understanding the HCP–patient relationship and whether there are any changes that might benefit both women and HCPs in the context of NIPT. HCPs have valuable perspectives on whether routinisation is happening, why they believe it is happening, and what the costs and benefits of it are. If studies have shown that HCPs believed the NIPT decision-making process could be routine, what were their reasons for feeling this way, and are those reasons justified? The answers to these questions are unknown, and more information about the attitudes of HCPs is needed to determine the extent to which routinisation might be an issue for NIPT.

## Research Methodology and Methods used

The overall project in which this study appears uses empirical ethics as the research methodology. This methodology attempts to use philosophical analysis and empirical data collection to argue toward normative considerations [30]. The study presented here comprises one of the empirical sections of that overall study, and the aim of this paper is to create a rich description of the context in which NIPT decision making occurs.

A gap in the peer-reviewed bioethics literature showed a dearth of qualitative studies focusing on UK HCPs that investigate NIPT in the interim period between 2012 and 2021 (unpublished manuscript). Therefore, this study used in-depth, semi-structured interviews [31], to investigate the views of healthcare professionals who offer NIPT. There was an effort to have a wide diversity of views collected within interviews. As such, a recruitment process was undertaken that might capture HCPs from different regions of the UK, from different professionals, and from a variety of professional practice sites. The aim of this study was not to find a representative sample, but rather to get a sense of all the different types of views that exist in the field. To sample such views, 20 UK HCPs were interviewed in 2020 about the ethical and professional issues related to NIPT use. While the country-wide NHS offer of NIPT began in 2021 in England, there were several NHS trusts in England that were already participating in a national NIPT rollout scheme, meaning those professionals were already offering NIPT in their workspaces at the time of interview. Therefore, the views expressed in interviews reflect participants from clinics that were already using NIPT in some capacity. In order to participate in this study, participants were required to have real experiences offering and/or providing counselling for NIPT in the UK.

Of the 20 participants, 12 were recruited from NHS clinics while 8 were recruited from private non-NHS-affiliated clinics. Their professions were sonography, genetic counselling, clinical genetics, midwifery, obstetrics, and healthcare counselling. The number of years of work experience in their respective roles ranged from 1 to 30 years with a median of 11 years. In the interview process, data saturation was assessed when few new ideas were being introduced in interviews. This was done at the discretion of the researcher. See Table 1 for some basic demographics of interview participants.

**Table 1:** Demographics of interview participants

Profession	Private	NHS	Total number
Obstetrician	2	2	4
Clinical geneticist	0	3	3
Genetic counsellor	1	3	4
Midwife	3	4	7
Sonographer	1	0	1
Healthcare counsellor	1	0	1
**Total**	**8**	**12**	**20**

Findings from the interviews were analysed using themes that arose from select literature written about the doctor-patient relationship [1–3]. This work builds on top of a critical analysis of themes that arise in the doctor-patient relationship (unpublished manuscript). Some of the themes found through that analysis include the balance of harms and benefits and the exchange of facts and values. From there, initial codes were developed using those literature-based themes, and transcripts were read line-by-line, using the initial codes to identify segments of text [32]. Secondary codes were then developed from the reading and analysis of the interview transcripts [32]. Once a list of secondary codes was defined, interview transcripts were re-read, and the new set of codes was applied to appropriate sections of text. From here, a thematic analysis of the collected dataset was carried out [33]. NVivo software was utilised in this process to manage data analysis. Coding trees and results were discussed with colleagues in bioethics who research NIPT and the ethics of decision making. The collected data and themes produced a rich description of the views and attitudes of HCPs who offer NIPT. This study looks at one aspect of that descriptive work—HCPs’ attitudes toward routinisation.

## Results

### HCPs’ Views and Attitudes on NIPT Routinisation

At least half of the HCPs who were interviewed for this study held a view that the NIPT decision-making process was routine, which will be exemplified by many of the quotes that follow. This was interpreted according to Rothman’s account of routinisation mentioned above. Analysis of the dataset also uncovered several themes that relate to NIPT routinisation. These themes include: (1) NIPT’s advance test characteristics did not preclude a routine offer; (2) some pregnant women seemed to agree to use NIPT without critically engaging in the decision-making process; (3) some clinical processes affected the NIPT offer; and (4) the testing pathway or healthcare system was structured in a way that might lead to routinisation. These themes will be explained in more detail below.

### Theme 1: NIPT’s Advance test Characteristics did not Preclude a Routine Offer

Some HCPs described the offer of NIPT as something they routinely carried out even though it was described as an ‘advanced screen’ and could provide more accurate results than initial screening tests. In one instance quoted below, NIPT being an advanced screen might not affect the offer so long as HCPs were mindful in the offer.

I think that we will be quite comfortable being able to offer it routinely because it is a test that we are familiar with, we’ve obviously been using it for a number of years now.… I think as long as again it’s done with that mindfulness that it is an enhanced screen, and we don’t make it out to be an all-singing, dancing test that will give them all the answers that they need, that we do explain it as screening test, then, I think if women make that choice, I feel that that choice is right.

Interview 10, NHS Deputy Screening Coordinator

It was therefore believed by some interviewees that a routine offer of NIPT could be unproblematic even when considering its high accuracy, so long as information about the test was communicated clearly, honestly, and in a way where women understood what information NIPT results could offer. Therefore, according to some, NIPT being routine was not necessarily problematic with regards to the provision of information.

Some HCPs recognised that NIPT might be offered in a routine manner, and this was at times connected to another characteristic of the test, its non-invasiveness.

Yes, it just requires a blood test, so I don’t see any problem… I think that makes it an easy test or an acceptable test for majority of the patients because it requires one more blood test and they have lots of blood tests in our clinic anyway… I think for them, it’s easier to say yes.

Interview 13, Private Obstetrician

This obstetrician held the belief that having more prenatal testing was beneficial to women, and having a straightforward decision-making process for NIPT was good for the well-being of pregnant women. Importantly, there seemed to be no issue with a routine offer of a physically non-invasive test according to some HCPs.

### Theme 2: Using NIPT Without Critically Engaging in the Decision-Making Process

One view held by some interviewees was that women might accept screening tests just because they are on offer. This view suggests that pregnant women might listen to what HCPs recommend, because they trust that the advice is good. Therefore, some HCPs suggested women might spend less time critically thinking about the testing and treatment options they described in consultations.

I think the majority of people take up the majority of tests and do what their doctors and healthcare professionals say, because they think that that’s in their best interest.

Interview 3, NHS Genetic Counsellor

While the quote above is about prenatal testing more generally, a similar attitude was held by some interviewees with regards to NIPT specifically. In this type of scenario described above, the decision-making process might be minimised without users of prenatal testing ever recognising the possibility of having a more drawn-out discussion. If there is any truth in what this genetic counsellor reports, this could indicate that routinisation might be a norm with regards to many screening tests and that NIPT routinisation is not extraordinary in this way.

### Theme 3: Clinical Processes were Described as Leading to Routinisation

According to some HCPs, the bundling of NIPT with other prenatal screening tests created a context where screening was a norm.

Because women have too many bloods done at the beginning of their pregnancy, you know, they have their booking, routine booking bloods. I think some of them, they just lump NIPT in with that.

Interview 4, NHS Genetic Counsellor

In the view described above, NIPT being routinely offered alongside many routine tests could equate NIPT to other screening tests, although they are inherently different. Here, the routinisation HCPs described was created by the clinical context and a bundled offer and not directly from the information HCPs provided.

Another clinical process, the tick-list of items to complete, created the perception that prenatal screening was routine according to some HCPs. A private midwife described the perceived experience of being on a screening pathway and how a tick-list could affect the way women might perceive some of the issues in the offer of NIPT.

But if you said the long list of, we’re going to have scans at this time, this time, this time, and a blood test at this time, this time, this time, we’re going to have the NIPT for this, and you’ll maybe, they’re found to be Rhesus negative, they want to have the NIPT test check to find out the chance of the baby’s blood group being negative as well. So yeah, there is the chance that it could all become lost in this big list of things they’re going to be having done. They’re all on a tick-list really of getting from one, to the next, to the next, which might hinder having enough consideration of some of the issues involved.

Interview 15, Private Midwife

Using such a tick-list could place constraints on the decision-making process according to the attitude held by this midwife and others. As an aside, tick-lists need not be ethically problematic—it is possible to design tick-lists to emphasise critical thinking and engagement. 

### Theme 4: A Routine Offer Created by the Testing Pathway

While a tick-list is a tool that might be used to facilitate decision making within the screening pathway, the screening pathway itself was described as another contextual feature that led to the routinisation of testing.

I suppose once you’re pregnant, you’re looked after, and there’s a pathway that you have appointments at all these gestations. They do all these checks on you, and it’s nice to know you’re being looked after… perhaps the women who don’t engage with healthcare very well or, I don’t know, low education, I don’t know, then the conveyor belt’s good, because then they’re looked after in terms of, you know, their own health and the baby’s health.

Interview 9, NHS Genetic Counsellor

If this is a true account of the testing pathway, it is not clear whether women have opportunities to critically engage with testing decisions as they move through such a pathway. From what some HCPs described, women might be prompted to make decisions without having fully considered them because of the testing pathway. This would be in-line with the definition of a routine offer understood in this paper. This type of streamlined pathway could arguably be a more practical way to offer NIPT since the time available to counsel women seems to be limited in some clinical contexts. Thus, if women had difficulties navigating their own pregnancy care, the testing pathway could create a supportive environment that looked after women. While a supportive environment might lead to greater well-being, it could also be seen as a ‘parental’ approach to healthcare, and this may affect the independence of women in the decision-making process.

### Positive Consequences of a Routine Offer

Even though there was mention of NIPT being routine, HCPs were not in agreement about whether the routinisation of NIPT is beneficial or problematic. Several HCPs, for example, discussed some positive consequences of a routine NIPT offer. One benefit of a routine offer with minimal information provided is that it could free up HCPs’ time, allowing professionals to care about other aspects of women’s healthcare. The view of having limited time and resources available to provide complex information was pervasive among interviewees, and a routine offer was justified by some as a more practical way to provide NIPT, given the kinds of resource constraints HCPs face. While timesaving would not normally be a significant enough factor to outweigh concerns regarding informed consent and patient autonomy, some HCPs believed routinisation that limits the amount of information was justified in the case of NIPT because most women will have negative (normal) screening results—the pretest information is rarely needed. This phenomenon was described by an NHS genetic counsellor.

In an ideal world every woman would have lots of counselling around all the options available and most women aren’t going to end up in that situation where they are given a bad news result… I think that so many women who don’t end up in that situation, then they don’t get the time to properly counsel through all these screenings, and what would you do with the results, because most women won’t end up in that position,… if they gave all that counselling time to every woman, I don’t think they’d have time for it.

Interview 9, NHS Genetic Counsellor

In an alternative viewpoint from the quote above, some HCPs believed providing less information could be viewed as unacceptable since this might disrespect women’s decision-making autonomy, and NIPT could subsequently lead to a major decision about invasive testing and termination. Both of these views were observed in the sample of professionals interviewed here and will be discussed below.

Another benefit to offering routine NIPT was that routinisation could lower the anxieties that might affect some women, according to some HCPs. One genetic counsellor suggested that presenting NIPT as a routine test could give the impression that the test is unremarkable.

I mean you could potentially raise a lot of people’s anxiety unnecessarily as well, so I think it’s hard to find a kind of happy middle ground. You don’t want to terrify everybody with having a routine test. So, the vast majority of them don’t actually need to worry.

Interview 3, NHS Genetic Counsellor

This type of routine, unremarkable presentation in the offer was seen as a benefit because it could lower the anxieties of pregnant women. As discussed above, most women will receive a negative (normal) result when using antenatal screening tests for Down’s, Edwards’, and Patau’s syndromes, because those conditions affect a small proportion of the population. Therefore, offering NIPT in a routine way could be a neutral experience for most women since they need not worry about a positive (abnormal) result. At the same time, the genetic counsellor quoted above was concerned about offering a test in a routine manner where women might not understand the implications of test results, and she did not know exactly how to balance providing sufficient information to women versus giving too much information that might lead to unnecessary anxiety.

### Possible Disadvantages of a Routine Offer

HCPs discussed two possible negative consequences of a routine NIPT offer. First, some HCPs who were interviewed here suggested that a routine offer of NIPT could limit the transmission of information. If true, this limiting of information could affect the quality of the decision-making process that follows. One obstetrician commented on the idea that NIPT is an easy test procedurally, which possibly leads to less information being provided to women about the test.

The problem with NIPT is it’s very quick, very easy, in that somebody could go along to a private clinic, you don’t know how much information they’ve given beforehand about the conditions let alone about the accuracy of the test.

Interviewee 12, Private Obstetrician

This obstetrician believed there was a clear connection between the ease of the test procedure and the little amount of information able to be provided by HCPs. If true, this could mean that in a quick offer, a minimal amount of information might be provided, and this could leave women with an informational gap.

Besides the provision of information, other HCPs believed that a routine offer might affect women’s opportunities to reflect on the decision to use NIPT. An NHS healthcare worker, for example, held the view that women might agree to test without understanding one’s options after results are returned.

Because it’s non-invasive, and because sometimes people don’t think through the implications of where would the next step be, some women can look to offer it as a package, and ‘yes, I’ll do that. I’ll have that, because it’s got to be good’ without thinking it through. But if you get a high-risk result, where are you going to take this next? Because you’ve already stepped on that train.

Interview 4, NHS Genetic Counsellor

If the above is true, it could mean that women might have limited opportunities to reflect on whether they wish to have NIPT. The concern from some HCPs was this this could lead to a situation where women are unprepared to act on test results once returned, putting them in a position where a decision has to be made in haste and potentially not in accordance with their values.

Additionally, some HCPs suggested their colleagues held routines that provided too little information about NIPT in the pre-test consultation, and this led to poor consequences for both HCPs and women in their opinion. These reports arose from interviewees who described their colleagues’ practice and were rarely a self-admission of any wrongdoing on their part. One example of this came from an NHS midwife.

A lot of them have anger towards their private company, because it wasn’t fully explained to them.… They went in for Down’s syndrome, Edwards’ and Patau’s, and all of a sudden, they were getting phone calls for these sex chromosome conditions, which, you know, they hadn’t even considered. And then, the next thing is, we’re picking up pieces for that.

Interview 14, NHS Midwife

Not only did some HCPs interviewed here believe the inadequate amount of information provided to women made their own clinical practice more difficult (as described in the quote above), there were also HCPs who indicated that this kind of offer might also lead to poor outcomes for pregnant women who used NIPT.

According to some interviewees, even if a routine offer did not typically lead to the kinds of cases where women were reportedly harmed or where HCPs were inconvenienced, it does not mean the offer was made ethically or in an appropriate manner. One private midwife shared this sentiment.

I suspect that at any given time there are people out there who have had a test with a provider who didn’t give good information and didn’t probably understand it very much themselves, and yes, they’ve had a low-chance result, and yes, their baby is born and is not affected by a trisomy, it doesn’t mean the test was conducted appropriately or that the woman understood what the test was or that if she’d got a different result it would’ve been alright.

Interview 11, Private Midwife

Even though there were few reports of negative consequences compared to many reports of unproblematic offers where limited information was routinely provided, some HCPs believed that routinely providing limited information to women disrespected women’s decision-making autonomy.

### Separate Consultation Suggested as a Solution

To counteract problematic, routine offers of NIPT, there was one solution discussed by several interviewees that might bring about more engagement. Having a separate NIPT consultation was suggested by some HCPs as a way to address some of the issues created by a routine NIPT offer. For example, one way to rectify the issue created by the bundling of tests was to separate out NIPT pre-test counselling from counselling for other routine blood tests.

If it’s not given its own pedestal and its own set of counselling, I could see how that could potentially be bundled into all the kind of routine blood tests that we do in pregnancy. And I think that’s a potential disadvantage, almost too easy and women may not be fully aware of what they’re being tested for. So, I think it’s important that it is not bundled in with everything else, that it is sort of separated out and not just perceived as a routine blood test.

Interview 6, NHS Midwife

HCPs reported that some of the consequences of deciding to use NIPT without considering the implications of the test seemed to include decisional regret on the part of the woman, which in some instances led to pregnant women experiencing distress. Some HCPs reported that the worst consequences of a routine NIPT offer could be avoided by providing NIPT as a separate consultation.

HCPs also believed that offering NIPT is a separate consultation from other prenatal tests seemed to lead to a more ethical offer, which potentially addresses the problematic aspects of routinisation of which Rothman’s speaks. One HCP suggested an offer with an extra step “means pregnant women have some agency and autonomy” (Interview 19, Healthcare professional). Another HCP wanted to see NIPT separated out from other tests because NIPT leads to decisions about anomalies that “may be incompatible with life” (Interview 15, Private midwife). These were some reasons why HCPs held the attitude that women might benefit from reflecting about NIPT at length ahead of a decision to use it. Several HCPs from both the NHS and from private clinics seemed to advocate for more time and separate appointments as a way to combat a routine NIPT offer, although it is not clear how realistic or feasible those ideas might be to implement.

## Discussion

A considerable number of HCPs interviewed here held the attitude that NIPT is being offered as a routine screening test in the UK. Commentators within the literature suggest that eliminating the potential physical risks associated with prenatal testing could make the decision to test easier for women [34]. At face value, a prenatal test that is less physically invasive could promote women’s well-being. However, this could make it easier for women to accept testing without thinking critically about it, and there are possible implications for whether this respects autonomy. In some of the data presented here, participants held the attitude that a routine offer could be seen as an indication by the HCP that the test was a recommendation and should be taken. This kind of concern was mentioned in the *Tentative Pregnancy* introduced above [7]. In a separate study, having a test offered routinely in a prenatal testing context as standard of care made it appear to be “a recommended thing”, according to a sample of women [35, p. 360]. Therefore, some women suggest that HCPs offering routine screening could be seen as a tacit approval of it.

There were reports here from interviewees that on occasion women were perceived to accept NIPT offers without fully considering the implications of that testing, although it cannot be stated what actually occurs in practice from this interview study. According to HCPs, the reasons why women might not fully consider the test were not based on the personal choice of the woman, but rather, the circumstances in which she found herself. Recall that Rothman says where options might be presented as routine, there might be less reflection on those options [7], and this might mean that patients are not prompted to think about and/or ask certain questions about a treatment or test. Rebecca Kukla similarly comments on aspects of the provision of information, routines, and the doctor-patient relationship. Kukla says that “when a medical intervention is represented as routine, a patient will rarely question the reasons for the intervention” (36, p. 31). This is problematic in Kukla’s view because it discourages the interrogation of options one is deciding upon. A routine offer could lead women to not being fully aware of all the conditions for which they test. This commentary is especially relevant in the context of the NIPT offer, because some HCPs here reported that women might decide to take a test without knowing the range of decisions that stem from possible results, which could undermine their decision. To complicate this further, suggesting that patients need only to engage more or be more active in the decision-making process—as some in the doctor-patient literature do [1–3]—could be difficult in response to the type of a routine practice context that was described by interviewees.

As noted above, some believed a routine NIPT offer could lead pregnant women to not be aware of all the conditions for which they test. It is not clear what kind of awareness might be needed for an ethical offer of NIPT. Interviewees mentioned that the pregnant woman ought to be able to explain in simple terms what screening test they are having and what the results might indicate. Specifically, some advocated that pregnant women should understand that NIPT is a screen that indicates the chance that the foetus might have one or more trisomy connections, and that it is not a test to, for example, ‘check that the baby is healthy’. Having pregnant women understand basic characteristics of NIPT and demonstrating that knowledge, even if simply, could demonstrate awareness about important features of NIPT according to some HCPs. However, one issue that arises might be how to assess a pregnant woman’s understanding since this could be considered gatekeeping behaviour that might disrespect one’s autonomy in a reproductive decision-making context.

Gerald Dworkin discusses some of these issues in his book *The Theory and Practice of Autonomy* [36], and his analysis has implications for whether decision makers ought to demonstrate knowledge. Dworkin suggests autonomous action is not only about freedom of action or acting in an unconstrained way, but rather, it is about whether people can authentically deliberate and reflect upon choices, desires, and values and make decisions that are in-line with those values. He continues by suggesting if a decision is considered in a situation where people do not critically think about their options, this does not lower the costs associated with that decision. It ignores those costs. He argues that decision-making costs being ignored could lead to hardship later on if, for example, one does not realise all of the facets of a decision one is making [36]. These decision-making costs being ignored might have led to some HCPs interviewed here to have feelings of frustration in their professional roles, because they felt they were caring for someone who was offered NIPT improperly as described in some of the quotes above. Other HCPs believed the situation created by ignoring decision-making costs could similarly disrespect pregnant women’s autonomy and cause them harm.

Dworkin’s focus on reflection and deliberation in the decision-making process is distinct from an account of autonomy where agents simply choose in an unconstrained way. The former seems to be more respectful of autonomous deliberation. Supposing Dworkin’s account exemplifies a more ethical decision-making process, however, it is not clear how to assess knowledge in an unproblematic way, especially in a reproductive decision-making environment. More work needs to be carried out to understand how knowledge might be assessed respectfully in the context of NIPT. This work might require more research with a combination of stakeholder engagement on this topic as well as consulting the ethics, sociology, and psychology literatures.

There is one remaining issue, however, about whether someone can autonomously choose to ignore the receipt of information. In a hypothetical situation, suppose there was certain knowledge that a pregnant woman was unaware of an important feature of NIPT that might affect the way she evaluates her choices, and the pregnant woman was also choosing to refuse further receipt of information related to her choices (for example, the pregnant woman is unaware of the fact that NIPT results could prompt an offer of an invasive, diagnostic test). Could this type of refusal where the HCP knows a patient’s knowledge base is incomplete be an autonomous refusal? If we take Dworkin’s account of autonomy into consideration with its focus on authentic deliberation and reflection, as opposed to freedom from constraints, then someone with incomplete knowledge is missing something critical in the decision-making process that could help them make a better decision. In some situations, then, HCPs might have a duty to present select information about NIPT even when it has been initially refused. What NIPT information falls in this category of being able to override a refusal? This is an artful judgement made by the HCP who understands the context and the patient. This type of overriding a refusal of information could help some women who might be less epistemically active, who might not be familiar enough with NIPT to know what questions are pertinent to ask. This conclusion coincides with a recent analysis that suggests neither a liberty- or duty-based account to autonomy can reasonably be used to support extreme views on the right not to know [37]. While their piece focuses on the debunking extremes cases of the right not to know one’s own medical information (for example, the refusal of test results), this thinking could also extend to include general, non-patient-centric information about tests and treatments since—like the former—these types of information can affect how people approach medical decision making. Assessing one’s knowledge base and informing individuals of important information may cause offence when insensitive language or a disrespectful approach is used. However, offering more information when done with tact, engagement, and thoughtfulness might better respect autonomy.

This issue becomes more complicated in private industry in the UK where HCPs reported NIPT might be offered as a bundle with other screening tests. In the view of some interviewees, this could lead women to be unaware of all the tests undertaken. Women not being told of the conditions their pregnancy was screened for is a historic issue that was documented within the peer-reviewed literature of the 1990s [12]. Some authors there suggested that a bundled offer of prenatal screening tests could lead women to not question what was being screened [12]. Press and Browner’s studies about AFP screening suggest that research participants “were able, therefore, to accept [a] ‘simple’ blood test without feeling the need to understand very much about it or ponder its implications” [12]. When this type of bundled package was then used in concert with specific phrasing that indicated the ease of the test, it seems to have further ignored the decision-making costs of which Dworkin speaks. A bundled offer might not necessarily disrespect women’s autonomy if she understands the offer and that is the kind of test she wants. One can imagine an example where a woman is offered routine NIPT, wishes the process to be quick, has been considering this for a long time, has read the literature on NIPT, and conveys basic knowledge about the test to the HCP. The fact that the offer was bundled does not inherently suggest autonomy was disrespected, as she could have made a conscious, well-reasoned argument about why she wanted NIPT bundled with other tests. For this offer to respect autonomy, however, there must be attention paid by HCPs to create an environment where all healthcare users can take an active and inquisitive stance where they might be able to deliberate and ask questions if they so choose. Creating this type of environment and stance is not conditional on whether tests are bundled.

Along with the issue of how to provide information, there was an issue about how much information HCPs can disclose given some of the time constraints they face. Clinicians are not able to offer information about every test they offer, especially where a single ‘test’ has a broad and inclusive focus—as some interviewees admitted—nor will future parents easily read through a booklet, pamphlet, or website that outline the specifics of a number of genetic anomalies. Older studies indicate women and couples might not be inclined to read these kinds of pre-test materials even when jargon has been simplified [12]. It is not clear whether an ethical offer of NIPT could include women being offered panel tests that screen for many conditions, as some HCPs described here, and whether women should only be given information about the conditions for which they screen positive. This seemed to be a practical way to achieve women’s consent and inform them of test results, because giving all of the information upfront about a multitude of genetic screens or in-depth information about a few well-known conditions did not seem to be feasible with the time constraints HCPs reported they are facing. Not providing information about the conditions screened for also seems counter to the conclusion reached above about the need to present important information. Kukla also considers the issue of time constraints and recommends that HCPs do not “add an extended conversation to the clinical encounter, but rather to shift the structure of the clinical visit so as to embed different expectations about patients’ role and needs as knowers, even if each part of this conversation is usually brief” [38, p. 34]. HCPs interviewed here reported they believed some pregnant women were not prepared for some of the test results returned to them and subsequently had difficult reactions to the situation created by not knowing about the conditions tested for early on in the decision-making process. This was reported to be worrying for the professionals caring after women. Kukla’s suggestion here highlights the importance of setting expectations for patients. Importantly, if enough forethought is put into crafting the specific language and counselling approach used, the setting of expectations that follows might be achieved briefly and within the confines of a time-constrained work schedule.

### Limitations

There are several limitations to this study. This study considered the views and attitudes of HCPs that were self-reported. The quotations highlight what HCPs suggest they say to pregnant women in consultations. For this reason, aspects of the decision-making process—and not the decision—form a large basis of the analysis here. This is because interviewing HCPs about the decision or action taken would be better suited for a study that includes pregnant women who had used NIPT. Moreover, with regards to what HCPs reported in interviews, it is possible that HCPs might have spoken differently in interviews with an academic researcher than they would have with a pregnant woman. Therefore, the conclusions arrived at were informed by interpretations of HCPs’ reported speech, and they should not be considered a true account of any clinical or healthcare context or about the ways in which women might be impacted by NIPT. Instead, the data provided here are subjective accounts of participants who provide NIPT, including what they remembered on the day of the interview and what information they were willing to share.

## Conclusion

This study explored UK HCPs’ attitudes about the offer of NIPT. HCPs in the UK held attitudes that the offer of NIPT is already occurring in a routine way. Whether the routinisation of NIPT is in fact occurring and how we evaluate that phenomenon will have to be based on future work. Interestingly, some HCPs reported that a routine offer of NIPT might be beneficial and others described it as problematic. This converges with literature on the routinisation of NIPT that suggests routines are not inherently good nor bad. The same conclusion is true with regards to whether routines might respect or disrespect autonomy. In both cases, more information is needed about the specific routine. Thus, this paper pushes against the notion that routinisation is inherently wrong, and it uses empirical data and directs us to the content of what has been routinised and pushes us to evaluate it. This suggests that whether a routine offer is ethical might have to be based on a case-by-case deliberation and weighing of principles in specific contexts where NIPT is offered.

## Data Availability

The datasets generated during and/or analysed during the current study are available from the corresponding author on reasonable request.
